# Validity of the PHQ-9 and PHQ-2 to screen for depression in nationwide primary care population in Latvia

**DOI:** 10.1186/s12991-018-0203-5

**Published:** 2018-08-02

**Authors:** Elmars Rancans, Marcis Trapencieris, Rolands Ivanovs, Jelena Vrublevska

**Affiliations:** 1 Department of Psychiatry and Narcology, Riga Stradins University, Tvaika Street 2, Riga, 1005 Latvia; 2 Institute of Philosophy and Sociology, University of Latvia, Kalpaka bulv. 4, Riga, Latvia

**Keywords:** Depression, Primary care, General practitioners, Validation

## Abstract

**Background:**

Depression is highly underdiagnosed in primary care settings in Latvia. Screening for depression in primary care is potentially an efficient way to find undetected case s and improve diagnostics. We aimed to validate both a nine-item and two-item Patient Health Questionnaire (PHQ-9 and PHQ-2) in the Latvian and Russian languages in primary care settings using a representative sample in Latvia.

**Materials and methods:**

The study was carried out within the framework of the National Research Program BIOMEDICINE to assess the prevalence of mental disorders at 24 primary care facilities. During a 1-week period, all consecutive adult patients were invited to complete the PHQ-9 and PHQ-2. Criterion validity was assessed against the Mini International Neuropsychiatric Interview (MINI).

**Results:**

There were 1467 patients who completed the PHQ-9 and the MINI. Overall, the PHQ-9 items showed good internal reliability (Cronbach’s alpha 0.81 for Latvian version and 0.79 for Russian version of the PHQ-9). A cut-off score of 8 or greater was established for the PHQ-9 (sensitivity 0.75 and 0.79, specificity 0.84 and 0.80 for Latvian and Russian languages, respectively). For the PHQ-2, a score of 2 or higher (sensitivity 0.79 and 0.79, specificity 0.65 and 0.67 for Latvian and Russian languages) detected more cases of depression than a score of 3 or higher.

**Conclusions:**

We suggest GPs ask patients to respond to the first 2 questions of the PHQ-9. If their score is positive, the patients should then complete the PHQ-9.

## Background

Depression is a common psychiatric condition that has widespread consequences both at the individual and societal level. It is among the leading non-fatal diseases globally [1]. Long term consequences of depression include reduced quality of life, risk of suicide, increased rates of hospital admission, increased risk for chronical medical conditions and stigmatization [2–5].

The WHO study on psychological problems in general health care across 14 countries found that 14% of individuals suffered from major depression [6]. Despite the fact that most care for depression is delivered by general practitioners, under-recognition of depression has been extensively described [7]. Depression is often under-detected in primary care: approximately 50% of GPs correctly identify depression cases, and even fewer, 34%, record it in their notes [8].

Despite rich data from studies of depression in primary care in Western Europe [9, 10], there still is a need for studies from Eastern Europe [11]. The best available data suggest that under-diagnosis of depression is particularly salient for Latvia, where the 12-month prevalence of depression has been estimated at 7.8%, but according to the data from the Latvian National Health Service, only 4423 unique patients have been diagnosed with a mood disorder by general practitioners (GPs) [12, 13].

Because of large estimates of underdiagnosed and undertreated depression in primary care, improved screening could reduce the burden of depression. Routine primary care screening can facilitate improvement of the diagnosis rates of adult depression and has been recommended by the US Preventive Services Task Force [14, 15]. However, it is notable that some national guidelines doubt the effectiveness of screening for depression [16].

It is essential that depression screening tools are reliable and valid to ensure that the results they generate are clinically correct [17]. There are numerous studies assessing the reliability and validity of depression screening tools, but there is currently no consensus on one particular screening tool to be used for depression screening across primary healthcare settings [18]. Moreover, to be acceptable in practice, it is essential that instruments are easy and quick to use [19].

The Patient Health Questionnaire-9 (PHQ-9) was developed as a depression screener for depression in primary care. The PHQ-9 is a self-rating instrument for depression developed in the late 1990s from the Primary Care Evaluation of Mental Disorders (PRIME-MD) [20] and based on the Diagnostic and Statistical Manual of Mental Disorders, Fourth Edition (DSM-IV) criteria for MDD [21]. This tool consisting of 9 items is known for its ease of completion for the patient, ease of scoring and interpretation, and public availability. It is used among racially and ethnically diverse populations. Respondents rate the scale items from 0 to 3 according to the frequency of their experience over the previous 2-week period (not at all, several days, more than half the days, or nearly every day). A cut-off score of ≥ 10 has been recommended for detecting cases of major depressive disorder (MDE) [21, 22]. Over 100 studies have examined the PHQ-9 in primary care [22]. Moreover, the PHQ-9 has been validated in medical populations [23–25], general populations [26–29] and psychiatric samples [30–34].

Of recent interest has been the use of fewer screening questions from the PHQ–9 [35, 36]. The PHQ-2 was developed for depression screening, with some evidence for a role in diagnosing depression [35, 37, 38]. These 2 questions, collectively known as the PHQ-2, ask about the frequency of the symptoms of depressed mood and anhedonia, scoring each as 0 (not at all) to 3 (nearly every day). The validation study of the PHQ-2 by Kroenke et al. included a sample of 580 primary care patients [35].

A valid depression screener in Latvian and Russian is important for Latvia because 61.8% of the population is Latvian, with the remainder being people from Russian language-speaking nations (Russia, Belarus, and Ukraine) [39, 40].

The aim of our study was to validate the PHQ-9 and PHQ-2 in Latvian and Russian languages using the Mini International Neuropsychiatric Interview (MINI) as the reference standard in a representative primary care sample.

## Materials and methods

The current study was carried out in 2015 within the framework of the National Research Program, BIOMEDICINE 2014–2017, a cross-sectional study to assess the prevalence of mental disorders in primary care settings in Latvia. The study recruited patients from 24 primary care facilities all over the country that covered all regions of Latvia. The survey was conducted in the two most commonly spoken languages in Latvia (Latvian and Russian). The programme was financed by the Latvian Ministry of Education and Science. The main aim of this programme is to develop new prevention, treatment, and diagnostic methods and practices, as well as biomedical technologies to improve public health in Latvia. The programme has existed since 2007 and comprises certain areas: cardiovascular and metabolic diseases, oncological diseases, and childhood and infectious diseases. Mental health was included in the programme for the first time.

During a 1-week period in each GP’s facility, all consecutive patients aged 18 years or older visiting a primary care physician with any health concerns were invited to participate in the study. Those who visited their GP for any administrative reasons were not included in the sample. No further restrictions on patient selection were implemented.

All consecutive patients were invited to complete the paper-and-pencil form of the PHQ-9 in the preferred language (Latvian or Russian) before seeing the GP, followed by interview with a structured socio-demographic questionnaire. All uncertainties and questions raised were explained by a psychiatrist. Both versions of the PHQ-9 in Latvia have been previously adapted and used in a nationwide general population study [41]. However, at that time, no cut-off score for Latvia was established, and a cut-off score ≥ 10 has been applied as recommended by Kroenke et al. [21]. In 2014, within the pilot project of the National Research Programme, BIOMEDICINE, that was conducted at 6 primary care facilities, the cut-off score of the PHQ-9 of ≥ 10 for both languages was established [42]. However, that study included validation of the PHQ-9 and not the PHQ-2 and had considerable limitations such as a small sample size that was not representative of the primary care population nationwide.

No more than 2 weeks after completing the PHQ-9, four psychiatrists who were blind to the PHQ-9 scores interviewed the respondents over the phone with the Mini International Neuropsychiatric Interview (MINI), Version 6.0.0. The MINI is a structured diagnostic interview that was validated by convergence with the Structured Clinical Interview for the DSM-III-R Patient Version (SCID-P) and the Composite International Diagnostic Interview (CIDI) and by expert professional opinion [43]. The good psychometric characteristics of the MINI, its ability to be administered rapidly, and its acceptability to patients made it a good choice for research purposes [44]. The MINI has been translated and adapted for both Latvian and Russian languages by the authorship holders and previously has been used in population-based study [13]. The MINI was used as the standard to determine the presence of major depressive episodes and was conducted over the telephone. Administering the MINI over the telephone is acceptable and was applied in other studies [45, 46]. In this study, all modules of the MINI were used. Participants diagnosed with depression or suicide ideations or attempt were referred for appropriate care.

This study was approved by the Ethics Committee of the Riga Stradins University, Riga, Latvia. The project was conducted in accordance with the Declaration of Helsinki and its subsequent amendments. All patients were enrolled after providing written informed consent. Neither participating family practices nor patients were compensated for their participation.

### Statistical analysis

The internal reliability of the PHQ-9 was assessed by Cronbach’s alpha coefficient. The criterion validity of the PHQ-9 and the PHQ-2 was assessed by receiver operating characteristic (ROC) analysis. The criterion validity of the PHQ-9 and the PHQ-2 was analysed in terms of sensitivity, specificity, and positive and negative predictive values for different cut-off scores. The Latvian and Russian versions of the MINI, which is used to diagnose major depressive disorder, were used as the criterion standard. Data analyses were performed in Stata version 14 (Stata Corp). A separate analysis was conducted for the Latvian and Russian languages.

## Results

In total, 1604 patients were invited to complete the PHQ-9 scale, and 1585 of them completed the PHQ-9. From those who completed the PHQ-9, 100 patients did not answer a telephone call three times and were excluded, and 1485 patients were interviewed with the MINI over the telephone. In the final analysis, 1467 patients (448 men and 1019 women) were included. The questionnaires of 18 patients had to be dropped out due to insufficient data quality.

The main characteristics of those who were included in the analysis are shown in Table 1. For both languages, a separate analysis was applied. According to the MINI, 10.2% (95% CI 8.7–11.8) of the whole population had current depression and 28.1% (95% CI 25.9–30.4) had experienced at least one depressive episode in the past. Current depression was found in 8.7% of those who completed the PHQ-9 in Latvian and 12.3% in Russian. The reliability (Cronbach’s alpha) for the Latvian version of the PHQ-9 scale was 0.82 and 0.79 for the Russian version.

**Table 1:** Characteristics of the total sample (*n* = 1467)

	Latvian	Russian
	% (*n*)	% (*n*)
Total	100 (912)	100 (555)
*Lifetime depression*	27.6 (252)	28.7 (159)
Past only depression	19.0 (173)	16.4 (91)
Current depression	8.7 (79)	12.3 (68)
*Gender*		
Male	30.7 (280)	30.3 (168)
Female	69.3 (632)	69.7 (387)
*Age groups*		
18–34	17.7 (161)	8.7 (48)
35–54	30.6 (279)	31.7 (176)
55–64	22.3 (203)	26.3 (146)
65+	29.5 (269)	33.3 (185)
*Education*		
Primary or less	16.2 (148)	15.4 (78)
Secondary	53.4 (487)	54.3 (310)
Higher than secondary	29.8 (272)	29.7 (164)
No answer	0.6 (5)	0.6 (3)
*Socioeconomic status*		
Above average	5.2 (47)	4.3 (24)
Average	71.3 (650)	60.9 (338)
Below average	23.5 (214)	34.8 (193)
No answer	0.1 (1)	0.0 (0)

**Fig. 1:**
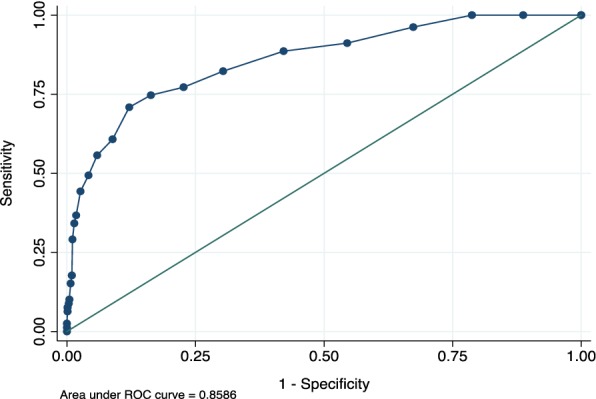
The receiver operating characteristic (ROC) curve of the Latvian version of the PHQ-9 versus the MINI for the major depression diagnosis

**Fig. 2:**
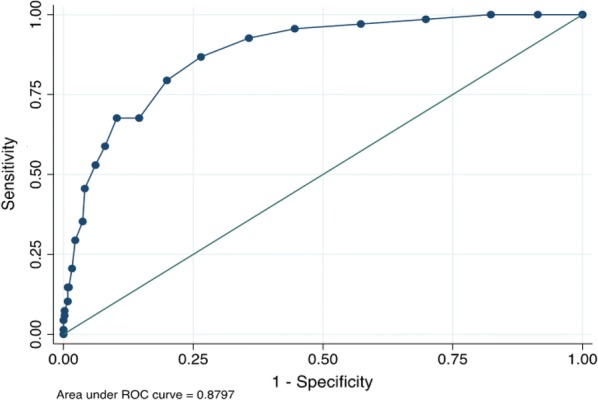
The receiver operating characteristic (ROC) curve of the Russian version of the PHQ-9 versus the MINI for the major depression diagnosis

The performance of the PHQ-9 was compared against the diagnosis of major depression as determined by the MINI, a reliable standard. The sensitivity, specificity, and likelihood ratio are presented separately for the Latvian and Russian languages in Tables 2 and 3, respectively. At a cut-off score of 8 or above, the sensitivity of the Latvian version of the PHQ-9 was 0.75, and the specificity was 0.84. For the Russian version of the PHQ-9, they were 0.79 and 0.80, respectively. The positive likelihood ratio was 4.57 for the Latvian version and 4.0 for the Russian version at this cut-off score. A cut-off score of 10 for the PHQ-9 Latvian language decreased sensitivity to 60.8% and increased specificity to 91.1%. A cut-off score of 10 for the PHQ-9 Russian language decreased sensitivity to 67.7% and increased specificity to 89.7%. The cut-offs chosen in the ROC curve analysis where the ones closer is to the upper left corner. ROC curve analysis (Figs. 1, 2) supported the criterion validity of the PHQ-9 in differentiating between patients with and without major depression (AUC = 0.86 for Latvian version and 0.88 for Russian version).

**Table 2:** Sensitivity, specificity, and likelihood ratios at various cut-off points of the Latvian version of the PHQ-9

PHQ-9 score	Sensitivity (%)	Specificity (%)	Classified (%)	LR+	LR−
≥ 6	82.3	69.6	70.7	2.71	0.25
≥ 7	77.2	77.3	77.3	3.40	0.29
≥ 8	74.7	83.7	82.9	4.57	0.30
≥ 9	70.9	87.9	86.4	5.84	0.33
≥ 10	60.8	91.1	88.5	6.84	0.43
≥ 11	55.7	94.1	90.8	9.47	0.47
≥ 12	49.4	95.8	91.8	11.75	0.53
≥ 13	44.3	97.4	92.8	16.78	0.57
≥ 14	36.7	98.2	92.9	20.39	0.64

**Table 3:** Sensitivity, specificity, and likelihood ratios at various cut-off points of the Russian version of the PHQ-9

PHQ-9 score	Sensitivity (%)	Specificity (%)	Classified (%)	LR+	LR−
≥ 6	92.7	64.2	67.8	2.59	0.11
≥ 7	86.8	73.5	75.1	3.28	0.18
≥ 8	79.4	80.1	80.0	4.00	0.26
≥ 9	67.7	85.4	83.2	4.64	0.38
≥ 10	67.7	89.7	87.0	6.59	0.36
≥ 11	58.8	92.0	87.9	7.34	0.45
≥ 12	52.9	93.8	88.8	8.59	0.50
≥ 13	45.6	95.9	89.7	11.10	0.57
≥ 14	35.2	96.3	88.8	9.55	0.67

We also performed validity analysis for both languages of the PHQ-2 against the MINI The sensitivity, specificity, LR+ and LR− for all possible PHQ-2 thresholds for both Latvian and Russian languages are presented in Tables 4 and 5. At the threshold ≥ 2, the PHQ-2 Latvian version correctly identified 78.5% of MINI cases (sensitivity) and 64.6% of non-cases of depression (specificity). The PHQ-2 Russian version correctly identified 79.4% of cases and 66.5% of non-cases. The positive likelihood ratio was 2.21 and 2.37 at this cut-off score for the Latvian and Russian languages, respectively. The PHQ-2 demonstrated moderate overall accuracy relative to the MINI for discriminating between cases and non-cases of depression, with an AUC of 0.79 for the Latvian version and AUC of 0.80 for the Russian version.

**Table 4:** Sensitivity, specificity, and likelihood ratios at various cut-off points of the Latvian version of the PHQ-2

PHQ-2 score	Sensitivity (%)	Specificity (%)	Classified (%)	LR+	LR−
≥ 1	89.9	40.7	45.0	1.52	0.25
≥ 2	78.5	64.6	65.8	2.22	0.33
≥ 3	55.7	89.9	87.0	5.52	0.49
≥ 4	36.00	94.2	89.4	6.59	0.66
≥ 5	22.8	98.0	91.5	11.16	0.79
≥ 6	15.2	98.4	91.2	9.73	0.86

**Table 5:** Sensitivity, specificity, and likelihood ratios at various cut-off points of the Russian version of the PHQ-2

PHQ-2 score	Sensitivity (%)	Specificity (%)	Classified (%)	LR+	LR−
≥ 1	94.1	38.2	45.1	1.52	0.15
≥ 2	79.4	66.5	68.1	2.37	0.31
≥ 3	58.8	87.7	84.1	4.77	0.47
≥ 4	45.6	92.8	87.0	6.34	0.59
≥ 5	27.9	96.3	87.9	7.56	0.75
≥ 6	19.1	98.2	88.5	10.34	0.82

## Discussion

The main aim of this study was to assess the validity of the PHQ-9 and the PHQ-2 and to establish a cut-off score to identify depression in the nationwide sample of patients attributable to Latvia visiting their GP because of health concerns. The screener was primarily developed for use in primary care settings and is the only questionnaire that has been tested in a primary care sample in Latvia.

Instruments that can be used in both screening and scaling modes have a particular advantage in that their weaknesses can be compensated by each other [47].

Within 18 studies performed with the PHQ-9, the prevalence of depression, as diagnosed by the gold-standard tests, ranged from 2.5 to 37.5% [48]. In our study, the point prevalence of depression was estimated at 10.2%, which is consistent with the findings from the other studies.

Despite the fact that the brief PHQ-9 is commonly used to screen for depression with 10 often recommended as a cut-off score, we found that a cut-off score of ≥ 8 on the PHQ-9 was the best at detecting depression in primary care patients in Latvia. Interestingly, the optimal cut-off points for major depression fall in the severity range of 5–9, as described by Kroenke et al. [21] for the category of patients with mild depressive symptoms. In the meta-analysis by Manea et al. [48], the PHQ-9 was found to have acceptable diagnostic properties for detecting depression for cut-off scores between 8 and 11. Its validity was supported by the AUC value that suggests a moderate accuracy of the questionnaire.

The pooled estimates of sensitivity and specificity for a cut-off score of 8 reported by Manea et al. [48] were 0.82 (95% CI 0.66–0.92) and 0.83 (95% CI 0.69–0.92), respectively. In our study, the rates of sensitivity and specificity for the Latvian language version were 0.75 and 0.83 and for the Russian language version were 0.79 and 0.80, respectively. In a study with primary care elderly patients, in which the criterion validity was evaluated by administering both the PHQ-9 and the MINI, the reported optimal cut-off score for major depressive disorder with the best validity characteristics was ≥ 7 (sensitivity 0.92, specificity 0.78) [49]. Our study showed lower sensitivity, but higher specificity. Although, we have also studied primary care populations, the comparison of the studies cannot be made easily. In our study, we included all patients who visit their GP because of medical concerns, but in the study by Lamers et al. [49], only the patients 60 years or older diagnosed with certain chronic medical disorders were included.

The sensitivity of screening instruments is considered good when their range is 0.79–0.97 and when their specificity is 0.63–0.86 [50]. Both languages of the PHQ-9 had relatively low sensitivity and acceptable specificity. The moderate specificity of the PHQ-9 for diagnosing major depression can be explained because it is possible to diagnose the disorder without having either of the two cardinal symptoms of major depression. As such, the summed score does not match perfectly with the MINI, which is a structured diagnostic interview based on DSM-IV criteria [51].

The internal consistency (alpha coefficient) of the PHQ-9 in this study was 0.82 for the Latvian version and 0.79 for the Russian version. For a self-report instrument to be reliable, it is suggested that Cronbach’s alpha be at least 0.70 [52]. However, it was lower than that from studies in the US (alpha coefficient = 0.79–0.89) [53, 54].

Recently, the PHQ-9 validation study in six primary care settings in Latvia was performed with a total sample size of 293 patients [42]. The estimated cut-off score was ≥ 10 with sensitivity 86.49% and specificity 89.36% for both languages. In the pilot project of the PHQ-9 validation, the PHQ-9 validity parameters were better than in this study. It is notable that the pilot study had considerable limitations. First, there was a small number of subjects. Second, not all Latvian regions were covered; therefore, the results cannot be representative. Third, the study was conducted by one interviewer. This study was conducted with a larger sample of patients and covered all Latvian regions and was performed by four mental health professionals who specialize in psychiatry and who were blind to the PHQ-9 and PHQ-2 results.

Our findings support the fact that an estimated cut-off score of 10 cannot be generalized across countries and populations.

The 2-question screener was sensitive for diagnosis of major depression when compared with the MINI with sensitivities of 0.90 and 0.94 for Latvian and Russian versions for thresholds of 1 or greater. Sensitivities for threshold 2 or greater comprised 0.79 for both language versions of the PHQ-2, and these sensitivities were acceptable. However, the specificities for threshold 1 or greater were not acceptable, but for threshold 2 or greater they were modest for both language versions of the PHQ-2: 0.65 for the Latvian version and 0.67 for the Russian version. At the most commonly used threshold ≥ 3 [35], the sensitivity for the Latvian and Russian versions was 0.56 and 0.59 and the specificity was 0.90 and 0.88, respectively. The finding that the score ≥ 2 was more successful at detecting depression is in accordance with similar finding reported by previous studies [55]. Another study to include a primary care sample (but not exclusively) reported a sensitivity of 0.83 and a specificity of 0.92 when the PHQ-2 (threshold score of 3 or higher) was compared with a health professional interview in 580 patients [35]. The patients who received the reference standard interview had to be contacted within 48 h of the screening interview. In our study, the reference standard was provided by the telephone within 2 weeks after the screening phase, which may have introduced bias into the results. A study conducted in older patients using the DSM-IV as a reference standard reported a sensitivity of 1.0 and a specificity of 0.77 for the PHQ-2 [56]. However, in this study, construct validity cut-off points were not reported. A study conducted in an outpatient clinic in Germany reported sensitivity and specificity was 78 and 79%, respectively, for major depression determined by a PHQ-2 score of 3 or more [37]. At a threshold score of 3 or higher and using a recognized reference standard, our sensitivity results for the PHQ-2 are generally not as high as those of other studies. This outcome can be explained as the result of a truly consecutive sample of patients in primary care, a reference standard that was administered not immediately but within 2 weeks after screening or even simply chance. Another of the limitations is its cross-sectional design; longitudinal studies are needed to establish the sensitivity to change. Inclusion of currently diagnosed and treated patients may increase bias in studies by inflating estimates of screening accuracy [57].

The strengths of this study are that all the patients were from primary care and they all received the MINI reference standard assessment. Our study included a large sample size, covered all Latvian regions and was conducted in urban and rural settlements and is representative to primary care in Latvia. Respondents were interviewed by four psychiatrists who were blind to the PHQ-9 estimates.

## Conclusion and implications for practice

In summary, the Latvian and Russian versions of the PHQ-9 and PHQ-2 have moderate psychometric properties for screening for major depression in general practice with a recommended cut-off score of 8 or greater for the PHQ-9 and 2 or greater for the PHQ-2. For GPs who wish to screen their patients for depression, we suggest they ask patients to respond to the first 2 questions of the PHQ-9 (i.e., the PHQ-2); if their score is positive (if they score 2 or more), the patients should then complete the PHQ-9.

In a study on the 12-month prevalence of depression and healthcare utilization in the general population of Latvia, certain risk factors for depression were identified [13], and these factors could be useful for GPs to identify the target population and initiate screening with the PHQ-2 and PHQ-9.

In this study, established cut-off points of the PHQ-9 and PHQ-2 together with the established risk factors for having depression in the study conducted in the general population [13] have been used within the framework of the National Research Programme, BIOMEDICINE, to develop diagnostic and treatment algorithms for depression in primary care in Latvia.

## Abbreviations

AUC: the area under curve
GP: general practitioner
LR+, LR−: likelihood ratio for positive and negative results
MINI: The Mini International Neuropsychiatric Interview
PHQ-9: The Patient Health Questionnaire-9
PHQ-2: The Patient Health Questionnaire-2
ROC: a receiver operating characteristic
US: The United States
WHO: The World Health Organization
